# Performance Evaluation of Deep Learning for the Detection and Segmentation of Thyroid Nodules: Systematic Review and Meta-Analysis

**DOI:** 10.2196/73516

**Published:** 2025-08-14

**Authors:** Jiayu Ni, Yue You, Xiaohe Wu, Xueke Chen, Jiaying Wang, Yuan Li

**Affiliations:** 1 Departement of Otolaryngology-Head and Neck Surgery, Affiliated Hospital of Hangzhou Normal University, No. 126, Wenzhou Road, Hangzhou, 310015, China, 86 15005812373; 2 Departement of Ultrasound, The First Affiliated Hospital of Xinjiang Medical University, Urumqi, China; 3 Departement of Ultrasound, Yangming Hospital Affiliated to Ningbo University, Yuyao, China; 4 Departement of Otolaryngology-Head and Neck Surgery, Hangzhou Normal University, Hangzhou, China; 5 Department of Otorhinolaryngology, Deqing Hospital of Hangzhou Normal University (The Third People's Hospital of Deqing), Huzhou, China

**Keywords:** thyroid imaging, artificial intelligence, diagnostic performance, sensitivity and specificity, systematic review, PRISMA, Preferred Reporting Items for Systematic reviews and Meta-Analyses

## Abstract

**Background:**

Thyroid cancer is one of the most common endocrine malignancies. Its incidence has steadily increased in recent years. Distinguishing between benign and malignant thyroid nodules (TNs) is challenging due to their overlapping imaging features. The rapid advancement of artificial intelligence (AI) in medical image analysis, particularly deep learning (DL) algorithms, has provided novel solutions for automated TN detection. However, existing studies exhibit substantial heterogeneity in diagnostic performance. Furthermore, no systematic evidence-based research comprehensively assesses the diagnostic performance of DL models in this field.

**Objective:**

This study aimed to execute a systematic review and meta-analysis to appraise the performance of DL algorithms in diagnosing TN malignancy, identify key factors influencing their diagnostic efficacy, and compare their accuracy with that of clinicians in image-based diagnosis.

**Methods:**

We systematically searched multiple databases, including PubMed, Cochrane, Embase, Web of Science, and IEEE, and identified 41 eligible studies for systematic review and meta-analysis. Based on the task type, studies were categorized into segmentation (n=14) and detection (n=27) tasks. The pooled sensitivity, specificity, and the area under the receiver operating characteristic curve (AUC) were calculated for each group. Subgroup analyses were performed to examine the impact of transfer learning and compare model performance against clinicians.

**Results:**

For segmentation tasks, the pooled sensitivity, specificity, and AUC were 82% (95% CI 79%‐84%), 95% (95% CI 92%‐96%), and 0.91 (95% CI 0.89‐0.94), respectively. For detection tasks, the pooled sensitivity, specificity, and AUC were 91% (95% CI 89%‐93%), 89% (95% CI 86%‐91%), and 0.96 (95% CI 0.93‐0.97), respectively. Some studies demonstrated that DL models could achieve diagnostic performance comparable with, or even exceeding, that of clinicians in certain scenarios. The application of transfer learning contributed to improved model performance.

**Conclusions:**

DL algorithms exhibit promising diagnostic accuracy in TN imaging, highlighting their potential as auxiliary diagnostic tools. However, current studies are limited by suboptimal methodological design, inconsistent image quality across datasets, and insufficient external validation, which may introduce bias. Future research should enhance methodological standardization, improve model interpretability, and promote transparent reporting to facilitate the sustainable clinical translation of DL-based solutions.

## Introduction

Thyroid cancer (TC) is the leading type of malignant tumor in the endocrine system. Over the past 3 decades, the global incidence of TC has steadily risen. Between 1980 and 1997, the prevalence was about 2.4%, while by 2009, it increased to 6.6% [1]. In clinical settings, the prevalence of TC ranges from approximately 19% to 68%. Furthermore, according to Bray et al [2], the global prevalence of TC ranks ninth, while its mortality is positioned sixth. This elevation may be closely tied to the development of diagnostic technologies and the improved rates of early disease detection. Evaluating the risk of TC in patients with thyroid nodules (TNs) is clinically important and helps to reduce health care costs and patient suffering. Among various diagnostic methods available, ultrasound imaging has emerged as the preferred diagnostic tool due to its simplicity, rapidity, and strong reproducibility. However, its interpretation is heavily dependent on the experience of radiologists, potentially leading to variability among various observers.

In order to address the above limitations, artificial intelligence (AI) is extensively applied in medical imaging today [3]. As a crucial branch of AI, machine learning (ML) technologies, particularly deep learning (DL) frameworks, have been rapidly developed, offering significant application potential and technical support for automated medical imaging tasks, like segmentation, detection, and classification [4]. DL enhances diagnostic accuracy and efficiency while fully and accurately capturing lesion information. It outperforms traditional segmentation methods in terms of feature extraction, generalization, and handling complex structures [5]. Nevertheless, due to the presence of high noise, the quality of ultrasound elastography images is relatively low, making automated segmentation and detection a challenging task.

As radiomics research gains increasing attention, a noticeable number of original studies [6-8] and meta-analyses [9-11] have been published across various medical fields, particularly in the field of thyroid disease. Despite being the standard imaging method for diagnosing TN and TC, ultrasound has been confirmed to have some limitations. However, radiomics shows the potential to offer more accurate and precise results in TN and TC diagnoses, with promising application prospects [12]. Despite the growing number of studies on DL-based methods for thyroid image analysis, there is still considerable variation in study design, dataset quality, model architecture, and performance evaluation metrics. In addition, many studies are limited by small sample sizes, insufficient external validation, and inadequate reporting transparency, which may reduce reproducibility and overestimate the diagnostic performance. Given these limitations, comprehensively assessing the diagnostic performance of DL algorithms is needed to offer an evidence-based understanding of their clinical use.

This meta-analysis thoroughly appraises the performance of DL models in the segmentation and detection of TC and TN images. The reasons for heterogeneity among studies are explored, and potential sources are discussed. The impact of dataset size, network architecture, and external validation on model performance has also been explored. In addition, the limitations of the included studies are discussed separately, providing guidance for deep investigations and promoting the advancement of DL in the clinical application of this disease.

## Methods

### Search Strategy

For this study, searches were carried out in PubMed, Cochrane, Embase, Web of Science, and IEEE databases, with the search timeframe extending from the inception of the databases to December 2024. All articles from the search were imported into EndNote for management. Duplicate records were excluded. The search was limited to articles published in English. Studies published earlier than 2018, reviews, conference abstracts, editorial reviews, and studies related to animal experiments were excluded. The complete search strategy for each database was created by a team of experienced clinicians and medical investigators. The detailed search strategy pertaining to the keywords and concepts included “Thyroid Nodule,” “Thyroid Cancer,” “Thyroid Lesion,” “Thyroid Tumor,” “Thyroid Neoplasm,” “Thyroid Carcinoma,” “Machine learning (ML),” “Deep learning (DL),” “Artificial Intelligence,” “Artificial Neural Network,” “External Validation,” and “Convolutional Neural Network.” We combined each concept’s medical subject headings and keywords with “OR” and then joined the concepts with “AND.” Specific search strategies were tailored for each database. Multimedia Appendix 1 provides a summary of the search strategy used in each database. This study was conducted in line with the PRISMA (Preferred Reporting Items for Systematic Reviews and Meta-Analyses) guidelines (the PRISMA 2020 checklist is provided in Checklist 1).

### Inclusion and Exclusion Criteria

The original studies were first screened by 2 independent investigators (JN and YY) using titles and abstracts. They reviewed the entire text afterward, following the inclusion and exclusion criteria. Any disagreements or differing opinions would be discussed and resolved with a third party (YL). Randomized controlled trials, cohort studies, case-control studies, and cross-sectional studies were included. We focused on studies that assessed the diagnostic performance of DL for TN detection and segmentation. Studies that reported diagnostic outcomes, like the area under the receiver operating characteristic curve (AUC) of summary receiver operating characteristics (SROC), concordance index, accuracy, pooled sensitivity, and specificity, were included. Imaging techniques used for TN and TC diagnoses, like ultrasound, computed tomography (CT), and magnetic resonance imaging, were included. Reviews, conference abstracts, case reports, letters to editors, comments, and unpublished gray studies were excluded. Studies that were not relevant to the inclusion criteria and were published in languages other than English were also excluded.

Reviews, conference abstracts, case reports, letters to editors, comments, and unpublished gray studies were excluded. Studies that were not relevant to the inclusion criteria and were published in languages other than English were also excluded.

### Data Extraction

The following data were extracted by 2 independent investigators (JN and YY): first author, publication year, sample size (including training and testing set sizes), mean or median age, indicator definition, algorithm, feature extraction, and selection details. In case of discrepancies, discussions with a third party (YL) were held to resolve them. Binary data for diagnostic accuracy were extracted directly into contingency tables, which included true-positives, false-positives, true-negatives, and false-negatives. These were then used to calculate pooled sensitivity, specificity, and other metrics. If a study presented multiple contingency tables for the same or various DL algorithms, they were assumed independent of each other.

### Quality Assessment

Two independent investigators leveraged the quality assessment of diagnostic accuracy studies using AI (QUADAS-AI) [13] and Review Manager (version 5.4) to appraise study quality. Four domains are appraised in the QUADAS-AI tool: (1) patient selection, (2) index test, (3) reference standard, and (4) flow and timing. Each domain was used to assess the risk of bias (ROB). Furthermore, the first 3 domains were also used to evaluate concerns about applicability.

### Statistical Analysis

The meta-analysis was implemented by means of the meta-analysis of diagnostic accuracy studies module in STATA (version 17). The pooled sensitivity and specificity, along with their 95% CIs, were appraised to quantify the predictive accuracy of radiomics. In addition, an SROC curve and AUC were generated to summarize diagnostic accuracy. We plotted the corresponding combined 95% CI and 95% prediction intervals around the mean sensitivity, specificity, and AUC estimates in the SROC plot.

To examine heterogeneity, a forest plot was created to display the pooled sensitivity and specificity, while the *I*^2^ and Q values were calculated. The *I*^2^ values were categorized as follows: 0%~25%, 25%~50%, 50%~75%, and >75%, indicating very low, low, moderate, and high heterogeneity between studies, correspondingly. A random-effects model was leveraged to pool the effect sizes from each study, addressing potential heterogeneity in true effect distributions. The model was specifically designed to aggregate sensitivity, specificity, and AUC values from a variety of studies. Its strength lies in its ability to effectively manage the differences between these metrics while recognizing their interconnections. In addition, we executed detailed subgroup analyses, including whether transfer learning (TL) and DL or ML algorithms were applied, to explore how different features and conditions affected the diagnostic performance of DL models.

### Ethical Considerations

The study was registered with the PROSPERO (International Prospective Register of Systematic Reviews; CRD42024599495). It followed the preferred reporting items for systematic reviews and meta-analyses guidelines [14]. For this study, no ethical approval or informed consent was needed.

## Results

### Literature Selection

**Figure 1.**
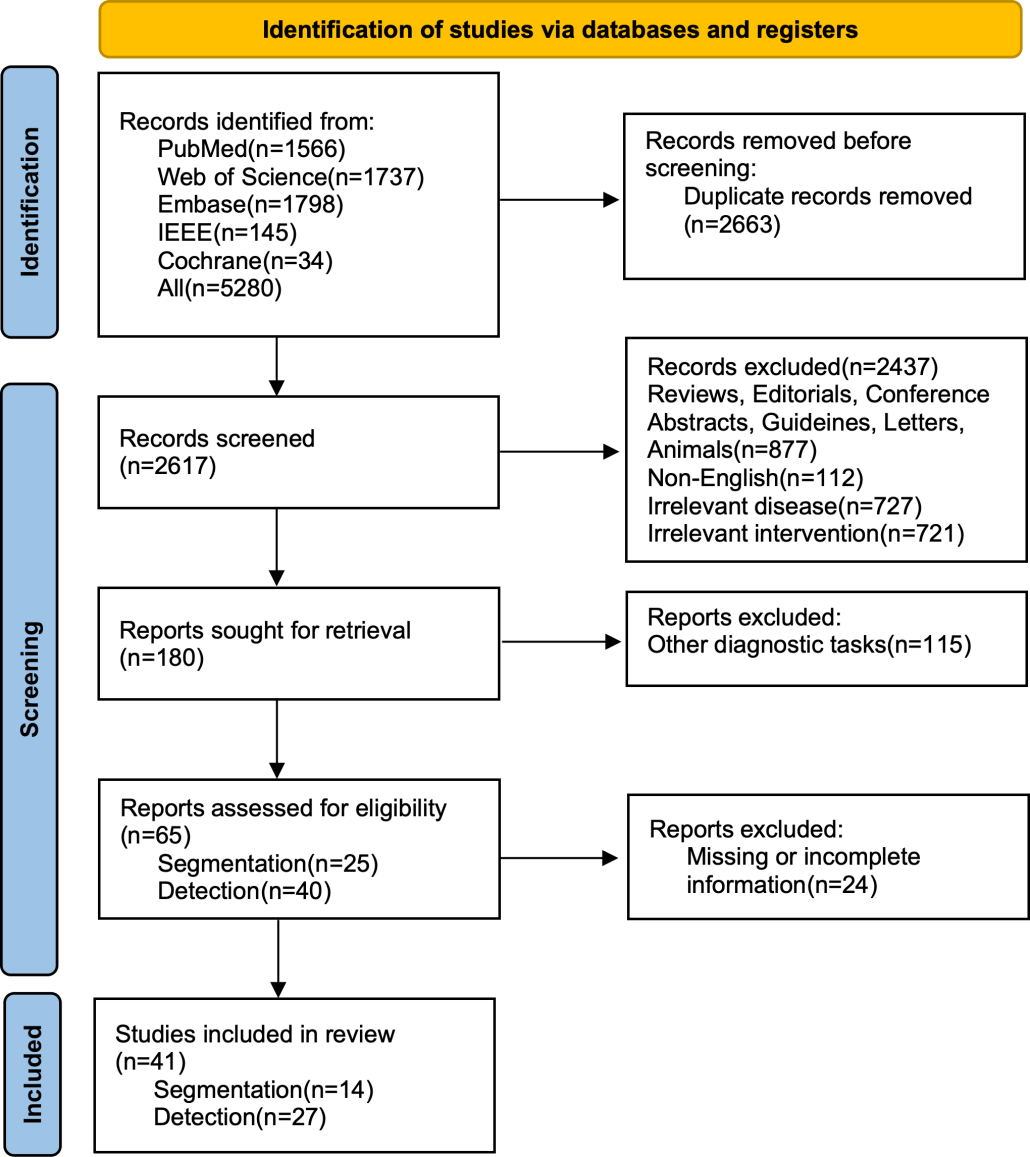
Study selection process following Preferred Reporting Items for Systematic reviews and Meta-Analyses guidelines. PRISMA: Preferred Reporting Items for Systematic Reviews and Meta-Analyses.

From the databases, 5280 articles were totally retrieved. Out of these articles, 2663 were reviewed based on their titles and abstracts after removing duplicates. Among these studies, 2576 were deleted for not fulfilling the inclusion criteria. Finally, 41 studies were included. Among these, 14 studies [15-28] centered on segmentation tasks, while 27 [29-55] studies focused on detection tasks (Figure 1).

### Study Characteristics

Before presenting the results of the meta-analysis, we briefly summarized the characteristics of the included studies. The 41 studies were published between 2018 and 2024, all of which were retrospective. Out of the 14 studies on segmentation tasks, 4 used data from private sources, while in studies on detection tasks, 2 used data from public sources. In terms of algorithm selection, all studies on segmentation tasks used DL algorithms, whereas in studies on detection tasks, 16 studies used DL algorithms, and 11 studies leveraged ML algorithms. In terms of medical imaging modalities, all studies on segmentation tasks extracted TN features from ultrasound images. Among studies on detection tasks, 2 studies used CT images for TN feature extraction, while one study used both ultrasound and shear wave elastography images. Regarding TL, 5 studies on segmentation tasks used TL. Ten studies on detection tasks also used TL, while the remaining studies on detection tasks merely mentioned it. Furthermore, none of the studies on segmentation tasks reported information on image quality. However, in studies on detection tasks, 13 studies excluded low-quality images (Tables S1-S3 in Multimedia Appendices 2-4).

### Algorithm Performance

#### Pooled Analysis

**Figure 2.**
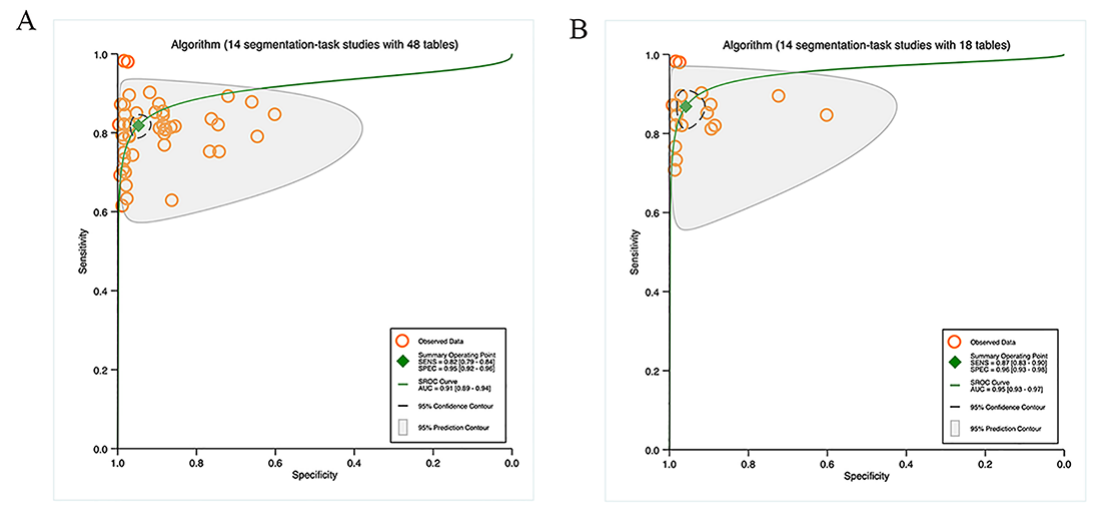
Pooled overall performance of algorithms: (A) Receiver operator characteristic curves of all studies on segmentation tasks (14 studies with 48 tables) and (B) receiver operator characteristic curves of studies on segmentation tasks reporting the highest accuracy (14 studies with 18 tables).

The 14 studies on segmentation tasks all provided sufficient data to create a contingency table for diagnostic performance. The hierarchical SROC curves for these studies (48 contingency tables) are depicted in Figure 2A . For all algorithms, the pooled findings indicated that the sensitivity and specificity were 82% (95% CI 79%‐84%) and 95% (95% CI 92%‐96%), and the AUC was 0.91 (95% CI 0.89‐0.94).

Since most studies on segmentation tasks used multiple algorithms to appraise diagnostic performance, the highest accuracy of these algorithms was appraised across 18 contingency tables. The pooled results demonstrated that the sensitivity and specificity were 87% (95% CI 83%‐90%) and 96% (95% CI 93%‐98%), and the AUC was 0.95 (95% CI 0.93‐0.97). Further details can be found in Figure 2B.

**Figure 3.**
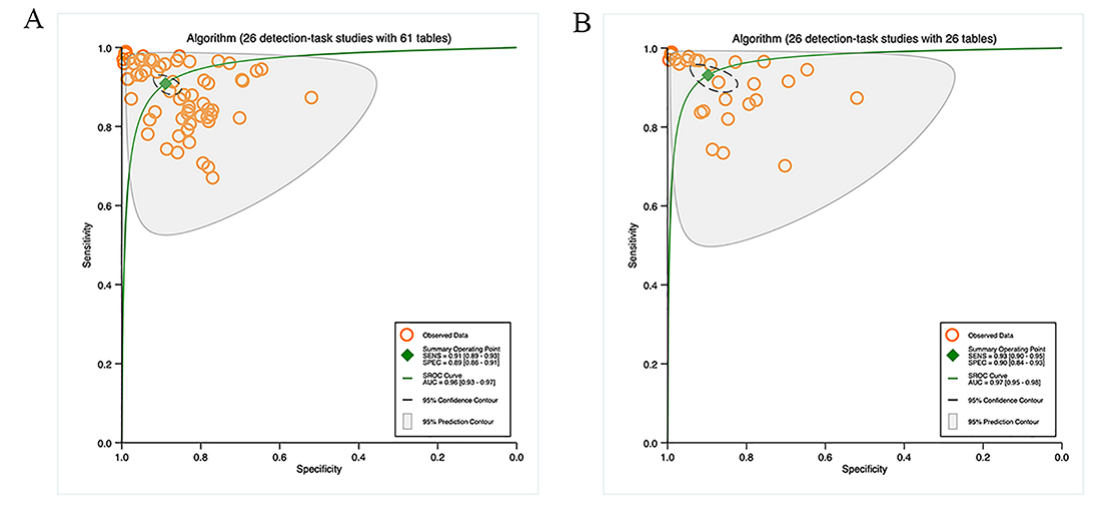
Pooled overall performance of algorithms; (A) Receiver operator characteristic curves of studies on all detection tasks (26 studies with 61 tables) and (B) Receiver operator characteristic curves of studies on detection tasks reporting the highest accuracy (26 studies with 26 tables).

In 26 studies on detection tasks, sufficient data were offered to generate a contingency table for diagnostic performance. Figure 3A illustrates the hierarchical SROC curves for these studies (61 contingency tables). The pooled results for all algorithms revealed that the sensitivity and specificity were 91% (95% CI 89%‐93%) and 89% (95% CI 86%‐91%), and the AUC was 0.96 (95% CI 0.93‐0.97).

The highest accuracy of various algorithms for detection tasks was appraised across 26 contingency tables. The pooled findings demonstrated that the sensitivity and specificity were 93% (95% CI 90%‐95%) and 90% (95% CI 84%‐93%), and the AUC was 0.97 (95% CI 0.95‐0.98). More details are available in Figure 3B.

#### Subgroup Analysis

##### Transfer Learning

**Figure 4.**
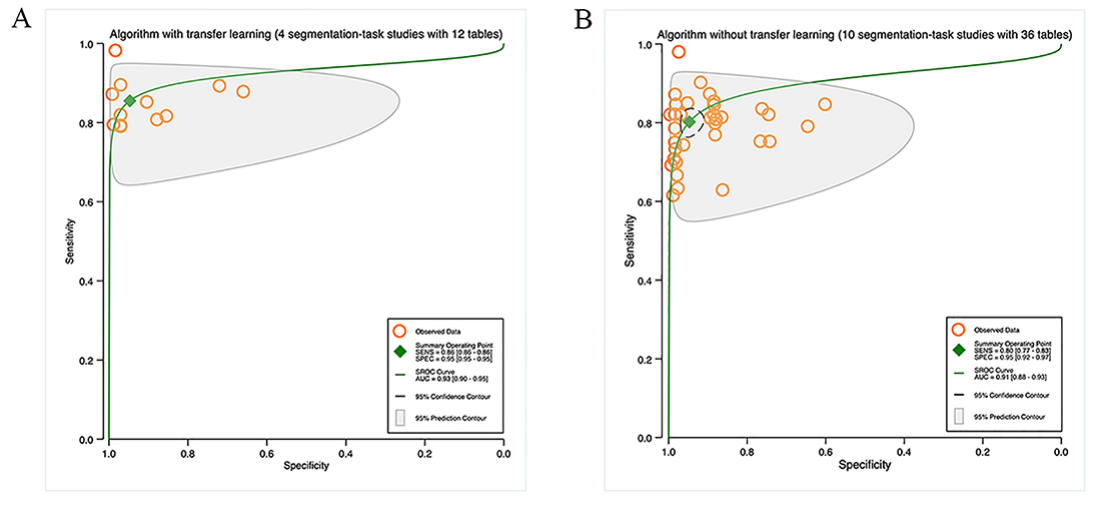
Pooled performance of algorithms with or without transfer learning: (A) Receiver operator characteristic curves of studies on segmentation tasks with transfer learning (4 studies with 12 tables) and (B) Receiver operator characteristic curves of studies on segmentation tasks without transfer learning (10 studies with 36 tables).

Four studies used TL for segmentation tasks, with 12 contingency tables. The pooled results indicated that the sensitivity and specificity were 86% (95% CI 86%‐86%) and 95% (95% CI 95%‐95%), correspondingly, with an AUC of 0.93 (95% CI 0.90‐0.95; Figure 4A). Ten studies on segmentation tasks did not mention the use of TL, with 36 contingency tables. According to the pooled results, the sensitivity and specificity were 80% (95% CI 77%‐83%) and 95% (95% CI 92%‐97%), and the AUC was 0.91 (95% CI 0.88‐0.93). Details can be found in Figure 4B.

**Figure 5.**
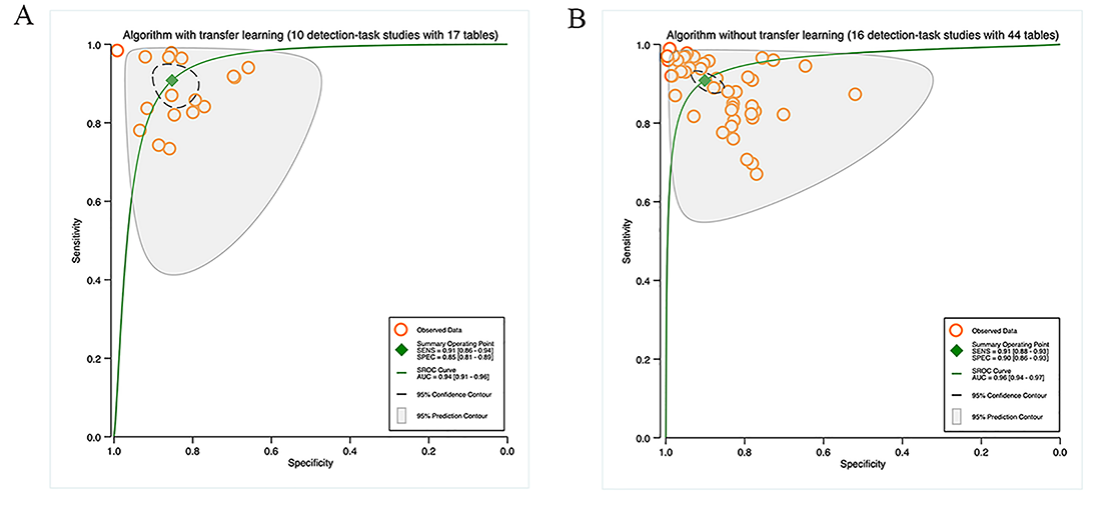
Pooled performance of algorithms with or without transfer learning: (A) Receiver operator characteristic curves of studies on detection tasks with transfer learning (10 studies with 17 tables) and (B) receiver operator characteristic curves of studies on detection tasks without transfer learning (16 studies with 44 tables).

Ten studies used TL for detection tasks, with 17 contingency tables. The pooled findings implied that the sensitivity and specificity were 91% (95% CI 86%‐94%) and 85% (95% CI 81%‐89%), correspondingly, with an AUC of 0.94 (95% CI 0.91‐0.96; Figure 5A). 16 studies on detection tasks did not mention the use of TL, with 44 contingency tables. The pooled results indicated that the sensitivity and specificity were 91% (95% CI 88%‐93%) and 90% (95% CI 86%‐93%), and the AUC was 0.96 (95% CI 0.94‐0.97). Details are available in Figure 5B.

##### DL Algorithms Versus Non-DL or ML Algorithms

**Figure 6.**
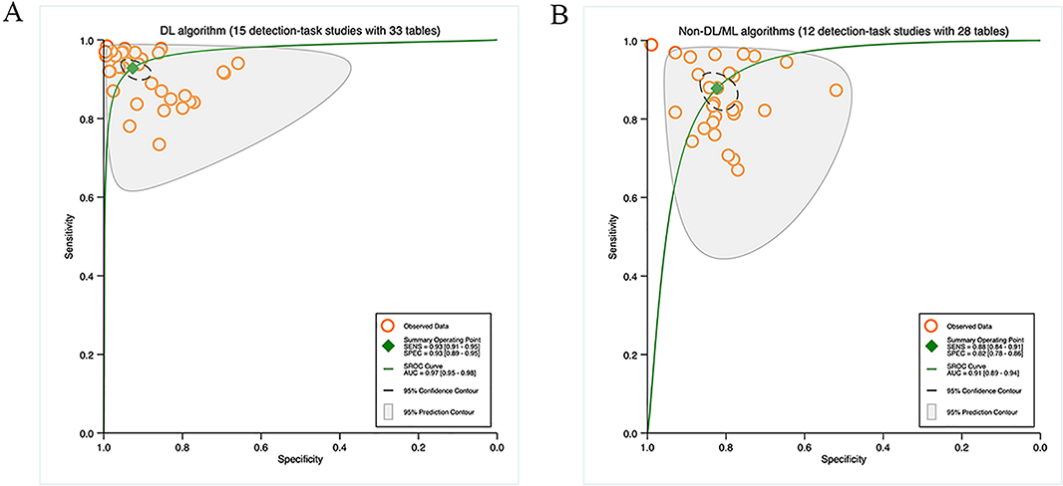
Pooled performance of deep learning algorithms or non-deep learning/machine learning algorithms: (A) Receiver operator characteristic curves for studies on detection tasks with deep learning algorithms (15 studies with 33 tables) and (B) receiver operator characteristic curves for studies on detection tasks with non-deep learning/machine learning algorithms (12 studies with 28 tables).

In 26 studies on detection tasks, the diagnostic performance of DL algorithms was compared with non-DL or ML algorithms, with 33 contingency tables for DL algorithms and 28 for non-DL or ML algorithms. According to the pooled results, the sensitivity was 93% (95% CI 91%‐95%) for DL algorithms and 88% (95% CI 84%‐91%) for non-DL or ML algorithms. The specificity was 93% (95% CI 89%‐95%) for DL algorithms and 82% (95% CI 78%‐86%) for non-DL or ML algorithms. The AUC was 0.97 (95% CI 0.95‐0.98) for DL algorithms and 0.91 (95% CI 0.89‐0.94) for non-DL or ML algorithms (Figures 6A and 6B).

##### Algorithms Versus Human Clinicians

**Figure 7.**
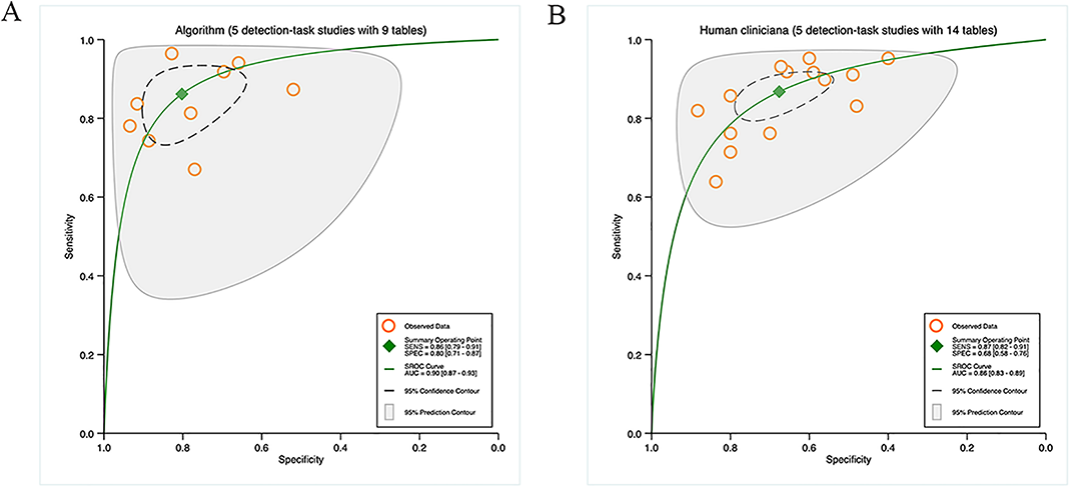
Pooled performance of algorithms versus human clinicians and human clinicians using the same sample: (A) Receiver operator characteristic curves of studies on detection tasks with algorithms (5 studies with 9 tables) and (B) receiver operator characteristic curves of studies on detection tasks with human clinicians (5 studies with 14 tables).

Five studies on detection tasks compared diagnostic performance between DL or ML algorithms and human clinicians using the same dataset, with 14 contingency tables for human clinicians and 9 for DL or ML algorithms. The pooled sensitivity was 86% (95% CI 79%‐91%) for algorithms and 87% (95% CI 82%‐91%) for human clinicians. The pooled specificity was 80% (95% CI 71%‐87%) for algorithms and 68% (95% CI 58%‐76%) for human clinicians. The AUC was 0.90 (95% CI 0.87‐0.93) for algorithms and 0.86 (95% CI 0.83‐0.89) for human clinicians (Figures 7A and 7B).

### Heterogeneity Analysis

**Figure 8.**
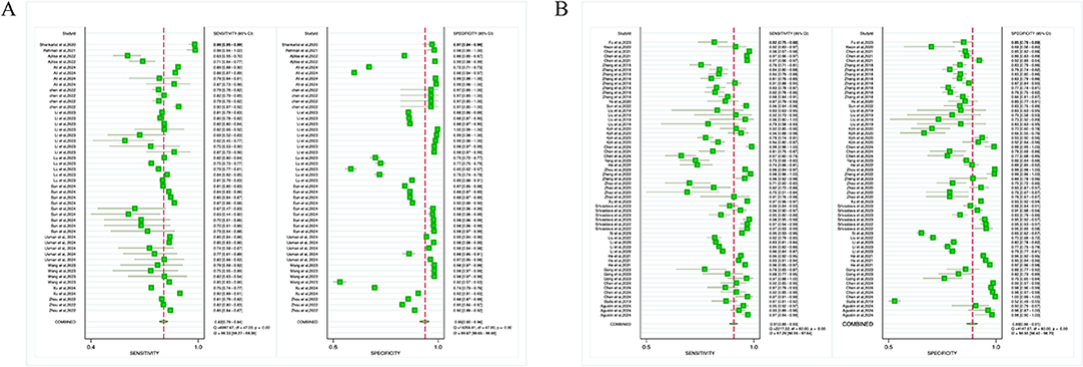
Summary estimate of pooled performance using forest plot: (A) Forest plot of studies on segmentation tasks (14 studies) and (B) forest plot of studies on detection tasks (27 studies). For a higher-resolution version of this figure, see Multimedia Appendix 6.

All included studies demonstrated that DL or ML algorithms were beneficial for TN segmentation and detection using medical imaging, in comparison with histopathological analysis. Nevertheless, considerable heterogeneity was noted. For studies on segmentation tasks, both sensitivity (*I*^2^=99.33%) and specificity (*I*^2^=99.67%) exhibited high heterogeneity (*P*<.0001; Figure 8A). For studies on detection tasks, sensitivity (*I*^2^=97.29%) and specificity (*I*^2^=98.55%) showed notable heterogeneity (*P*<.0001; Figure 8B).

Deek’s funnel plots generated using STATA 17.0 were used to assess publication bias. No publication bias was noted in studies on segmentation tasks (*P*=.09) and detection tasks (*P*=.50), even though the studies were widely distributed around the regression line (Figure S1a-b in Multimedia Appendix 5). To determine the sources of the extreme heterogeneity, subgroup analyses were conducted.

#### Transfer L

The results of heterogeneity analysis for the subgroup analysis based on the application of TL were as follows: studies on segmentation tasks with TL (sensitivity: *I*^2^=99.05%, specificity: *I*^2^=99.59, *P*<.0001), studies on segmentation tasks without TL (sensitivity: *I*^2^=99.32%, specificity: *I*^2^=99.65, *P*<.0001), studies on detection tasks with TL (sensitivity: *I*^2^=98.38%, specificity: *I*^2^=95.09, *P*<.0001), and studies on detection tasks without TL (sensitivity: *I*^2^=96.08%, specificity: *I*^2^=98.89, *P*<.0001; Figure S2-S3 in MultimediaAppendices 7  8).

#### DL Algorithms Versus Non-DL or ML Algorithms

The results of heterogeneity analysis for the subgroup analysis based on the application of DL algorithms were as follows: studies on detection tasks with DL algorithms (sensitivity: *I*^2^=98.17%, specificity: *I*^2^=98.24, *P*<.0001), and studies on detection tasks with non-DL/ML algorithms (sensitivity: *I*^2^=96.72%, specificity: *I*^2^=98.25, *P*<.0001; Figure S4 in Multimedia Appendix 9). Nevertheless, the source of heterogeneity did not stem from specific subgroups, as *I*^2^ values remained high. Therefore, we could not infer whether TL and algorithm models likely influenced the performance of algorithms for segmenting and detecting TN.

### Quality Assessment

The quality of the included studies was appraised by means of the QUADAS-AI (Figure S5a-b in Multimedia Appendix 10). A thorough evaluation of each item, based on the ROB domain and applicability concerns, is presented in Figure S6a-b in Multimedia Appendix 11.

#### Studies on Segmentation Tasks

For the patient selection domain, 3 studies were rated as unclear ROB due to unreported inclusion or exclusion criteria or improper exclusions. Regarding the index test domain, only 1 study was classified as having high or unclear ROB due to the absence of a predefined threshold, while the others were deemed to have low ROB. Three studies were deemed to have unclear ROB due to inconsistencies in reference standards. There was no mention of whether the threshold was determined in advance and whether blinding was implemented. For the flow and timing domain, 5 studies were considered to have high or unclear ROB as their authors did not mention whether an appropriate time gap was maintained or whether the same gold standard was used.

#### Studies on Detection Tasks

Regarding the patient selection domain, 9 studies were considered to have high or unclear ROB unreported inclusion or exclusion criteria or improper exclusions. In terms of the index test domain, 6 studies were deemed to have high or unclear ROB due to the absence of a predefined threshold, while the remaining studies were considered to have low ROB. Only 1 study was rated as unclear ROB due to inconsistencies in the reference standard. The predetermination of the threshold and the implementation of blinding were not mentioned. Regarding the flow and timing domain, 11 studies were classified as high or unclear ROB because their authors did not specify whether an appropriate time gap was maintained or if the same gold standard was leveraged.

## Discussion

This meta-analysis evaluates the performance of DL models in the segmentation and detection of TC and TN images. The results uncover that the pooled sensitivity, specificity, and AUC for segmentation tasks are 86%, 95%, and 0.93, respectively. For detection tasks, the combined sensitivity, specificity, and AUC are 91%, 85%, and 0.94, respectively. Some of the studies also compare the performance of DL models with that of the clinicians in image interpretation. The results reveal that the 2 are closer in terms of accuracy. This implies that AI technologies might assist in TN diagnosis. DL has high diagnostic accuracy for recognizing benign and malignant TN in imaging.

TN is frequently observed in clinical settings. The prevalence of TC has been rising steadily on a global scale in recent years [56]. In clinical settings, accurately identifying the few malignant nodules with clinical significance among the many benign TNs is challenging. This is crucial for determining which patients require biopsy or surgical removal, ultimately reducing health care costs and patient suffering. Hence, a reliable and noninvasive approach to assess TN is urgently needed. In clinical settings, radiologists preliminarily rely on visual standards for diagnosis, like size ratio, size, calcification, structure (single or multiple), borders, and echogenic characteristics (hyperechoic, isoechoic, or hypoechoic). Furthermore, due to differences in technical expertise, subjective experience, and physical condition, radiologists may interpret thyroid ultrasound images differently [57]

Through image recognition technology, AI can support physicians in making fast, precise, and efficient clinical decisions [58,59]. For example, DL models can recognize tumor boundaries and even predict the type and growth rate of tumors by learning from extensive image data. In addition, lymph node metastasis is closely linked to the local recurrence, distant metastasis, and staging of TC, providing remarkable guidance for the development of the surgical plan. Thus, the integration of AI into clinical practice demonstrates favorable performance in modern healthcare. Convolutional neural networks (CNNs) are regarded as one of the most advanced algorithms, applied in segmentation [60], detection [61], and classification [62] of TN. Ma et al [63] have used a CNN model for TN segmentation. Furthermore, Li et al [64] have developed a more improved Faster R-CNN based on CNN for TN detection. However, given the vastness and complexity of biomedical data, it is crucial to conduct rigorous testing on it [65].

After carefully selecting studies on related topics, it is found that ML algorithms exhibit excellent performance in medical image–based segmentation and detection of TN, demonstrating comparable or even superior performance to human clinicians. This study appraises the performance of distinct algorithm types (including DL or ML) based on different task types, considering the use of TL, as well as the performance under various levels of ROB. Furthermore, potential sources of heterogeneity between studies are identified based on the above subgroups. More importantly, study quality and ROB are critically assessed using the adapted QUADAS-AI [13] assessment tool. This is the strength of this study, providing better guidance to future related studies. This study seeks to identify accurate and reliable detection methods in the segmentation and diagnostic detection of TN.

By systematically searching the relevant studies, 4 systematic reviews and meta-analyses on ML algorithms for TN in medical imaging are found. Cleere et al [66] focus on the application of radiomics in TN diagnosis. Their study does not explicitly analyze the symmetry of the funnel plots and may miss studies with negative results, leading to an overestimation of the performance of imaging histology. The accuracy of imaging histology is highly dependent on ultrasound image quality and segmentation accuracy. Nevertheless, image standardization or quality control measures are not discussed in detail in their paper. Two studies investigate the accuracy of DL algorithms in diagnosing the benign and malignant characteristics of TN through ultrasound imaging. According to Zhu et al [11] and Zhong et al [9], the VGGNet (a CNN) model and S-Detect both demonstrate high sensitivity and specificity in differentiating between benign and malignant TN. Nonetheless, the greater level of heterogeneity and the relatively low quality of the samples render their results less persuasive. Besides, Zhao et al [67] are the first to appraise the diagnostic performance of the computer-aided diagnosis system for TN. However, their study only appraises computer-aided design (CAD) systems and does not cover a wider range of imaging histology methods. Furthermore, it fails to provide an in-depth discussion of the algorithmic differences between CAD systems. Based on the results of the above studies, this study has conducted a targeted comparative analysis and optimized the above deficiencies. Next, a detailed explanation of the comparison between DL algorithms and non-DL or ML algorithms will be provided, aiming to offer more substantial support and references for theoretical development and practical applications in this field.

This study reveals that DL algorithms are capable of segmenting and detecting TN using medical images. The 6 studies on detection tasks included mention comparisons between ML algorithms and clinicians, as well as comparisons between ML algorithms and clinicians working in conjunction with ML algorithms. The results indicate that DL algorithms demonstrate performance comparable with that of clinical physicians, and in certain respects, they may even exhibit superior capabilities. Nevertheless, it is essential to critically assess some problems of this evidence. In fact, both the judgments made solely by ML and those made by clinicians are subject to certain avoidable research biases. Comparing the diagnostic performance between AI and human clinicians is challenging. AI systems may have lower sensitivity and even higher error rates. Thus, we should not hastily conclude that AI has outpaced clinicians, as both have their respective advantages. Hence, it is more feasible to combine ML with clinicians and use ML as a supportive tool for diagnostic decision-making in clinical research. With continuous development and improvements, AI is expected to have an even greater impact on TN diagnosis in the future by optimizing algorithms and increasing training data.

The studies included in this article are all retrospective, leading to notable methodological flaws. In clinical settings, accurately obtaining test data is crucial for interpreting model performance. In the 41 included studies, only Koh et al [49] conducted external validation using multicenter data. Most studies on detection tasks are conducted in single centers without external validation, limiting the generalization ability of algorithm models. The risk of overfitting has also increased, leading to decreased reproducibility and affecting the reliability of the study. The ability of models to generalize is a key consideration in practical clinical applications, especially in environments with high data heterogeneity. Thus, we cannot adequately assess the performance of models in different populations and imaging sources. Most included studies conduct cross-validation internally, either through random or nonrandom methods. Using internal datasets to validate the model is more likely to be homogeneous and may lead to an overestimation of diagnostic performance, especially in private datasets where investigators may remove images that are difficult to detect. Strict external validation is required when designing AI-related diagnostic studies. Furthermore, in the 14 studies on segmentation tasks, only 4 are based on non–open access datasets. Public datasets are beneficial for reducing health care costs and making it easier to compare the performance of various algorithms and models, but there may be discrepancies in image quality, such as resolution, noise levels, and the accuracy of annotations. These differences may also have an impact on the generalization ability of models and performance outcomes. In addition, studies using public datasets generally do not specify inclusion and exclusion criteria, potentially leading to images with limited relevance and representativeness, increasing heterogeneity between studies, and affecting the reliability of the results. Furthermore, although 41 studies meet the inclusion criteria for the study, only half of the studies could be used to generate the specified contingency tables. Numerous studies use evaluation metrics like the Dice similarity coefficient, *F*_1_-score, and Jaccard index. However, these metrics are not comprehensive and may provide insufficient information to fully construct a contingency table when used alone. Therefore, in certain conditions, it is necessary to compute, supplement, or derive the missing components of the confusion matrix to ensure a comprehensive and accurate evaluation. In future studies, clearly defined metrics should also be carefully considered [68].

The sources and types of medical images are diverse, encompassing clinical laboratory reports, clinical images, and information derived from medical devices. The quality of the images notably affects the training and prediction capabilities of DL. In practical applications, factors such as image resolution, noise, and annotation quality should be considered, and appropriate preprocessing and augmentation measures should be taken to improve the performance and generalization ability of models. Due to the limited number of public datasets for TN, the public datasets used in the studies included are relatively homogeneous, such as the DDTI dataset [69] and the TN3K dataset [70]. Despite conducting an Egger linear regression test based on data extracted from the 41 studies, no evidence of publication bias is noted. However, the absence of prospective studies and the presence of negative results in studies may introduce potential biases. Therefore, there is a need for more high-quality studies, like prospective studies and clinical trials, to strengthen the existing evidence base [71]. It has been suggested by investigators that using synthetic data to augment experiments can overcome the limitations posed by restricted data [72].

Although DL algorithms have demonstrated promising diagnostic performance in the detection and segmentation of TNs, certain limitations persist within the included studies. First, most of the studies do not provide sufficient detailed information on model parameters or fine-tuning strategies, limiting our ability to evaluate the robustness, reproducibility, and generalizability of the models across different clinical scenarios. Second, few studies have reported on the computational cost, especially in terms of computational resources and processing time in the inference phase. These are especially critical for the deployment of models in real clinical settings, as processing speed and hardware efficiency directly affect their usability. In the absence of information on inference elapsed time or hardware requirements, it is difficult to determine whether these models are suitable for embedding in routine diagnostic processes. In addition, some of the studies exhibit potential biases, including selection bias and validation bias. These biases may arise from the inclusion of data from only a specific institution, a specific image quality, or a single population, which may limit the model’s ability to generalize to a wide range of populations. At the same time, insufficient external data validation further affects the judgment of its clinical applicability. This is in line with the retrospective data issues mentioned by Chu et al [73] in their meta-analysis of retinopathy of prematurity diagnosis.

The inclusion criteria for this study cover a wide range of study designs (like randomized controlled trials, cohort studies, case-control studies, and cross-sectional studies). It is worth noting that all the studies ultimately included are retrospective, which reduces methodological heterogeneity to a certain extent. Nevertheless, it also precludes us from carrying out subgroup analyses or adjustments with respect to study type. Consequently, it limits our ability to perform a subgroup analysis or adjustments for the performance of the model across different study contexts of a comprehensive assessment. In addition, retrospective studies are inherently more susceptible to selection bias and information bias, which may interfere with the estimation of model diagnostic performance. Therefore, caution should be exercised when interpreting the combined results and emphasizing the need for more prospective, high-quality studies in the future to validate the robustness and generalizability of the current findings.

We preliminarily believe that DL algorithms are capable of automatically segmenting and detecting TN, demonstrating high sensitivity and specificity comparable to that of clinical clinicians. Furthermore, these algorithms possess noticeable potential in the segmentation and detection of TN based on medical imaging. Nonetheless, it should also be noted that this finding comes from studies with relatively low methodological quality, which inevitably leads to an overestimation of the accuracy of the algorithms. The study design of ML-based segmentation and detection of TN still needs further refinement. In addition, AI application in medical diagnosis also raises important ethical and social issues, like transparency of algorithms, attribution of responsibility in case of diagnostic errors, and privacy protection of patient data [74,75]. Future research should pay more attention to these aspects in order to realize the responsible application of DL models in the clinic.

## Supplementary material

10.2196/73516 Multimedia Appendix 1 Search terms and search strategy.

10.2196/73516 Multimedia Appendix 2 Study design and basic demographics.

10.2196/73516 Multimedia Appendix 3 Methods of model training and validation.

10.2196/73516 Multimedia Appendix 4 Indicators, algorithms, and data sources.

10.2196/73516 Multimedia Appendix 5 Publication bias.

10.2196/73516 Multimedia Appendix 6 Summary estimate of pooled performance using forest plot: (A) Forest plot of studies on segmentation tasks (14 studies) and (B) forest plot of studies on detection tasks (27 studies).

10.2196/73516 Multimedia Appendix 7 Summary estimate of pooled performance using forest plot.

10.2196/73516 Multimedia Appendix 8 Summary estimate of pooled performance using forest plot.

10.2196/73516 Multimedia Appendix 9 Summary estimate of pooled performance using forest plot.

10.2196/73516 Multimedia Appendix 10 Quality assessment of diagnostic accuracy studies-2 summary plot.

10.2196/73516 Multimedia Appendix 11 Risk of bias and concern of applicability for each item in included studies.

10.2196/73516 Checklist 1 PRISMA (Preferred Reporting Items for Systematic Reviews and Meta-Analyses) 2020 checklist.

## Abbreviations

AI: artificial intelligence
AUC: area under the receiver operating characteristic curve
CAD: computer-aided design
CNN: convolutional neural network
CT: computed tomography
DL: deep learning
ML: machine learning
PRISMA: Preferred Reporting Items for Systematic reviews and Meta-Analyses
PROSPERO: International Prospective Register of Systematic Reviews
QUADAS-AI: quality assessment of diagnostic accuracy studies using AI
ROB: risk of bias
SROC: summary receiver operating characteristics
TC: thyroid cancer
TL: transfer learning
TN: thyroid nodule
