# Supplementary material for: Performance Evaluation of Deep Learning for the Detection and Segmentation of Thyroid Nodules: Systematic Review and Meta-Analysis
Source: J Med Internet Res. 2025 Aug 14;27:e73516. doi: 10.2196/73516 (PMC12352704; doi:10.2196/73516)

**Supplementary Note 1: Search terms and search strategy**

Database：Pubmed、Cochrane、Embase、Web of science、IEEE

**1. Thyroid Nodule [Mesh]**

Thyroid Lesion
Thyroid Tumor
Thyroid Neoplasm
Thyroid Carcinoma
Thyroid Cancer

**2.Machine learning [Mesh]**

Deep learning

artificial intelligence

artificial neural network

External validation

Convolutional Neural Network

Pubmed: n=1566

(Thyroid Nodule OR Thyroid Lesion OR Thyroid Tumor OR Thyroid Neoplasm OR Thyroid Carcinoma OR Thyroid Cancer) AND (machine learning OR Deep learning OR artificial intelligence OR artificial neural network OR External validation OR Convolutional Neural Network)

web of science: n=1737

Thyroid Nodule (Topic) or Thyroid Lesion (Topic) or Thyroid Tumor (Topic) or Thyroid Neoplasm (Topic) or Thyroid Carcinoma (Topic) or Thyroid Cancer (Topic) and Preprint Citation Index (Exclude—Database)AND(machine learning (Topic) or Deep learning (Topic) or artificial intelligence (Topic) or artificial neural network (Topic) or External validation (Topic) or Convolutional Neural Network (Topic) and Preprint Citation Index (Exclude – Database)

Embase: n=1798


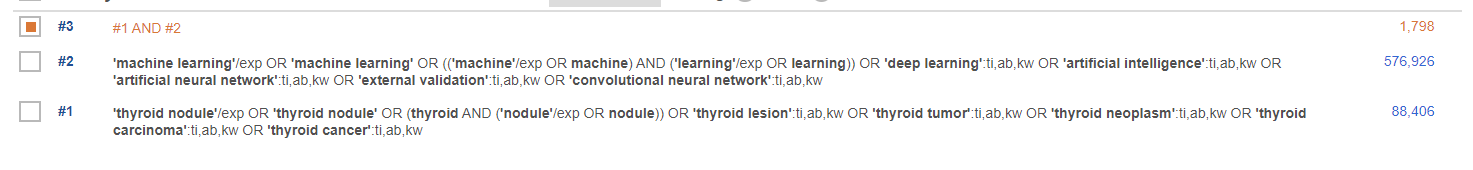


IEEE: n=145

(Thyroid Nodule OR Thyroid Lesion OR Thyroid Tumor OR Thyroid Neoplasm OR Thyroid Carcinoma OR Thyroid Cancer) AND (machine learning OR Deep learning OR artificial intelligence OR artificial neural network OR External validation OR Convolutional Neural Network)

cochrane: n=34


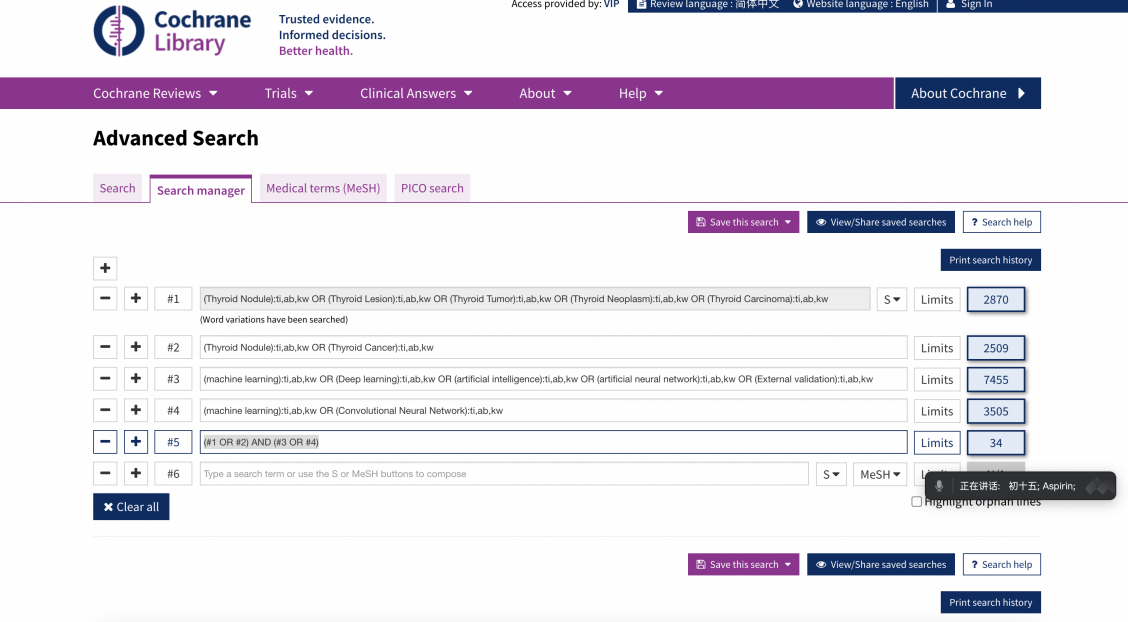

Supplement: Multimedia Appendix 1 [file jmir-v27-e73516-s001.docx]
