# Supplementary figures and images for: Performance Evaluation of Deep Learning for the Detection and Segmentation of Thyroid Nodules: Systematic Review and Meta-Analysis
Source: J Med Internet Res. 2025 Aug 14;27:e73516. doi: 10.2196/73516 (PMC12352704; doi:10.2196/73516)

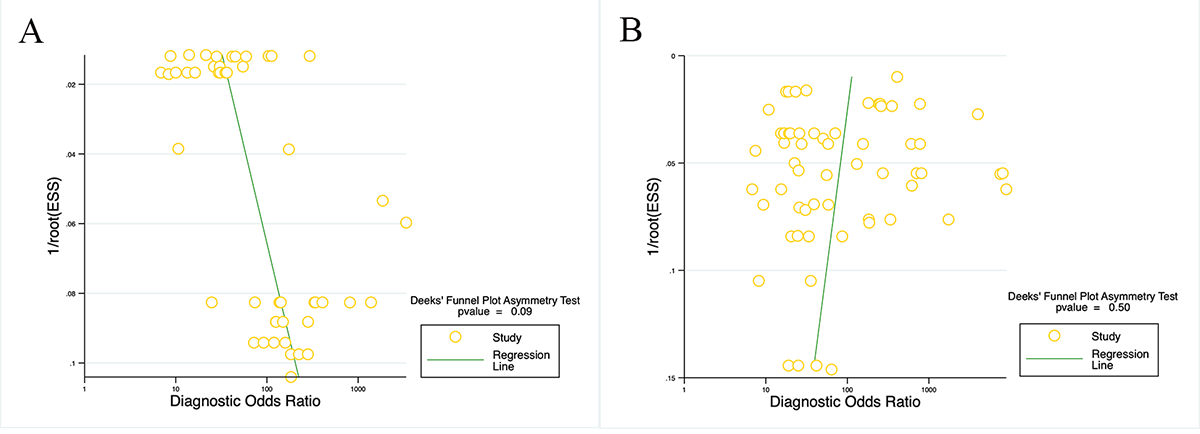

Supplement: Multimedia Appendix 5 [file jmir-v27-e73516-s005.png]

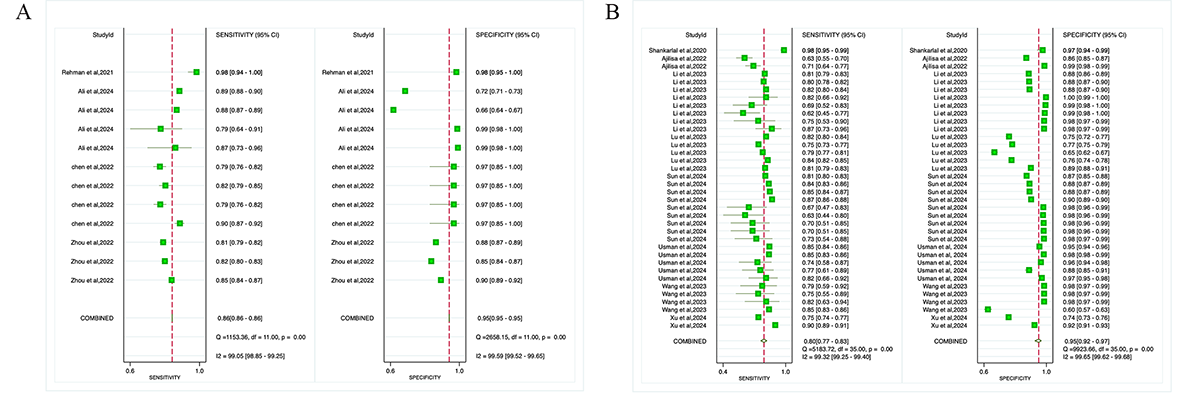

Supplement: Multimedia Appendix 7 [file jmir-v27-e73516-s007.png]

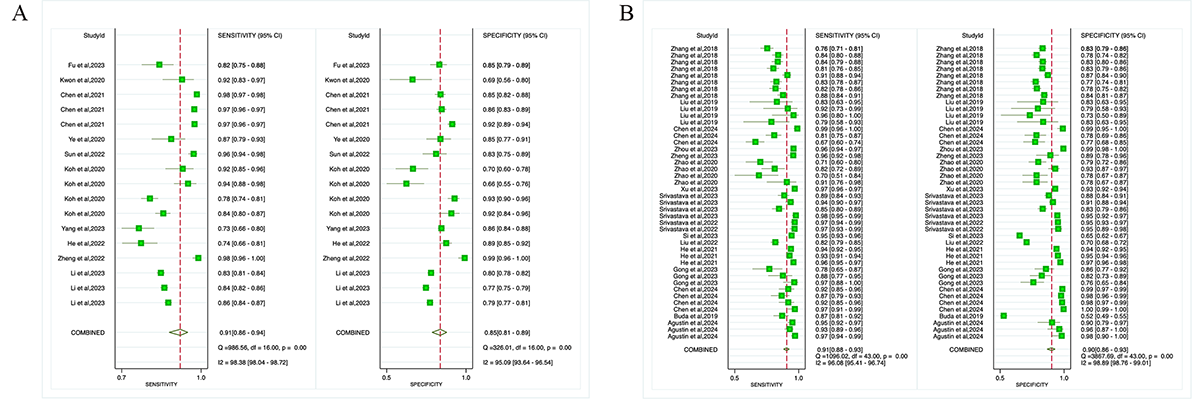

Supplement: Multimedia Appendix 8 [file jmir-v27-e73516-s008.png]

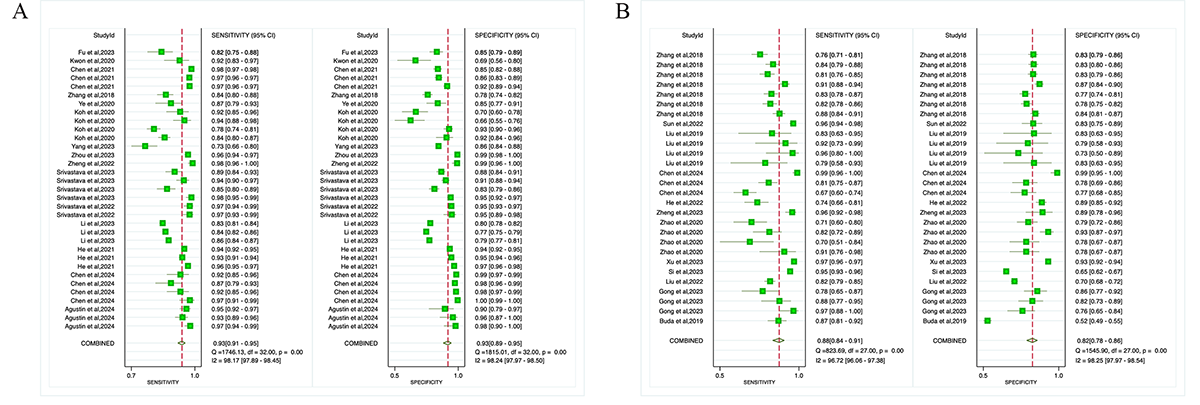

Supplement: Multimedia Appendix 9 [file jmir-v27-e73516-s009.png]

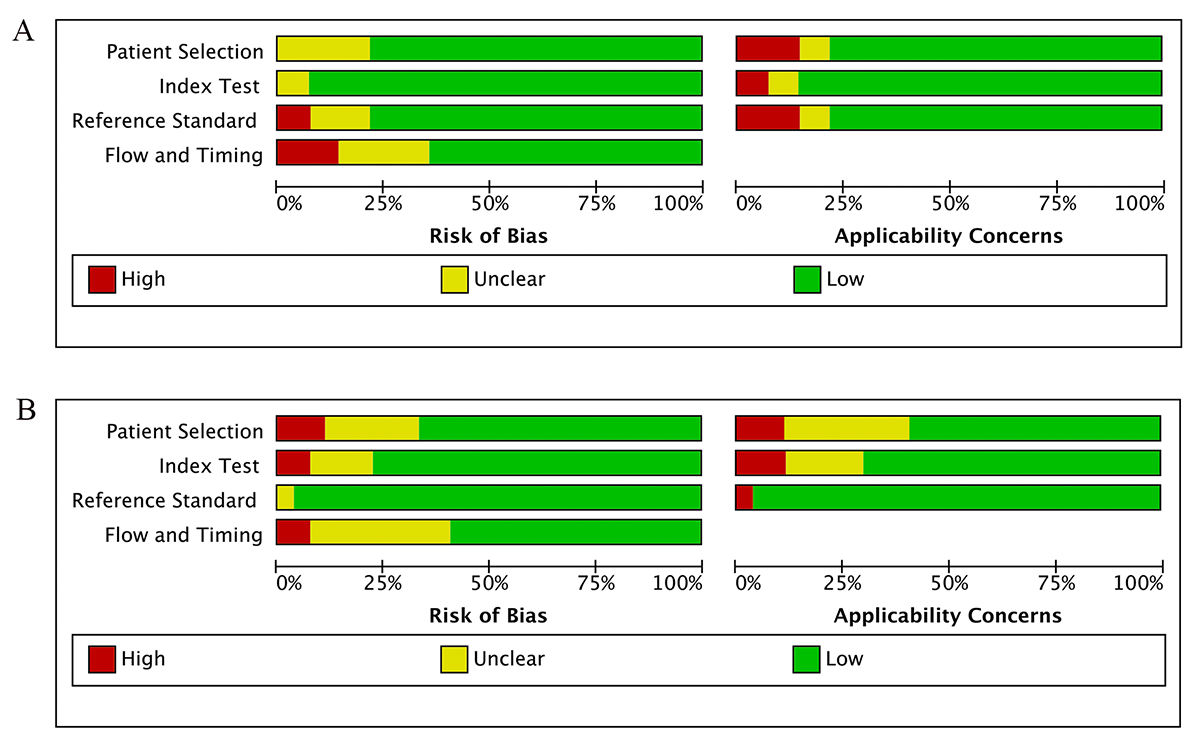

Supplement: Multimedia Appendix 10 [file jmir-v27-e73516-s010.png]

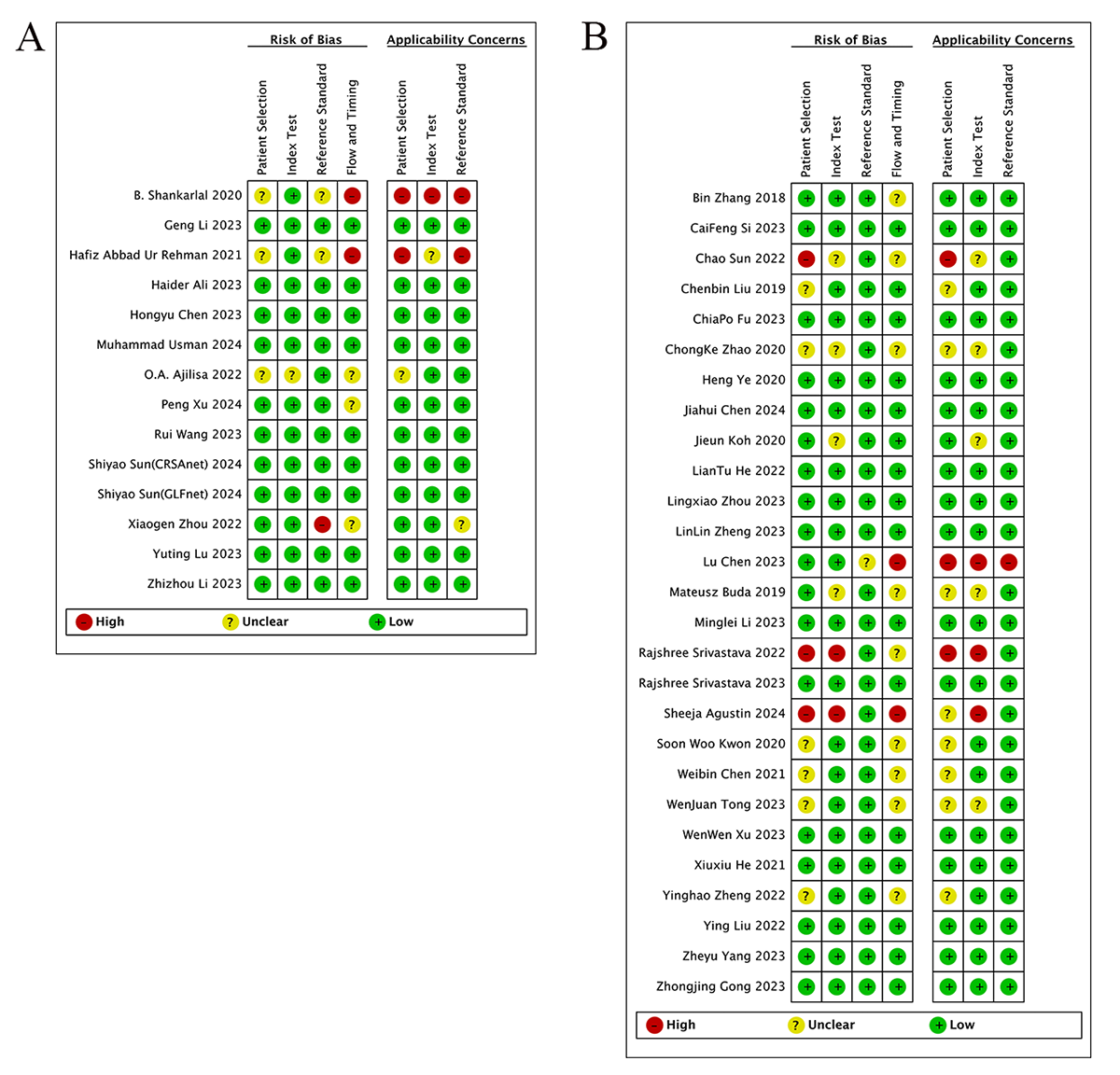

Supplement: Multimedia Appendix 11 [file jmir-v27-e73516-s011.png]
