# Supplementary material for: Performance Evaluation of Deep Learning for the Detection and Segmentation of Thyroid Nodules: Systematic Review and Meta-Analysis
Source: J Med Internet Res. 2025 Aug 14;27:e73516. doi: 10.2196/73516 (PMC12352704; doi:10.2196/73516)

**Figure 8**. Summary estimate of pooled performance using forest plot: (A) Forest plot of studies on segmentation tasks (14 studies) and (B) forest plot of studies on detection tasks (27 studies).

A.


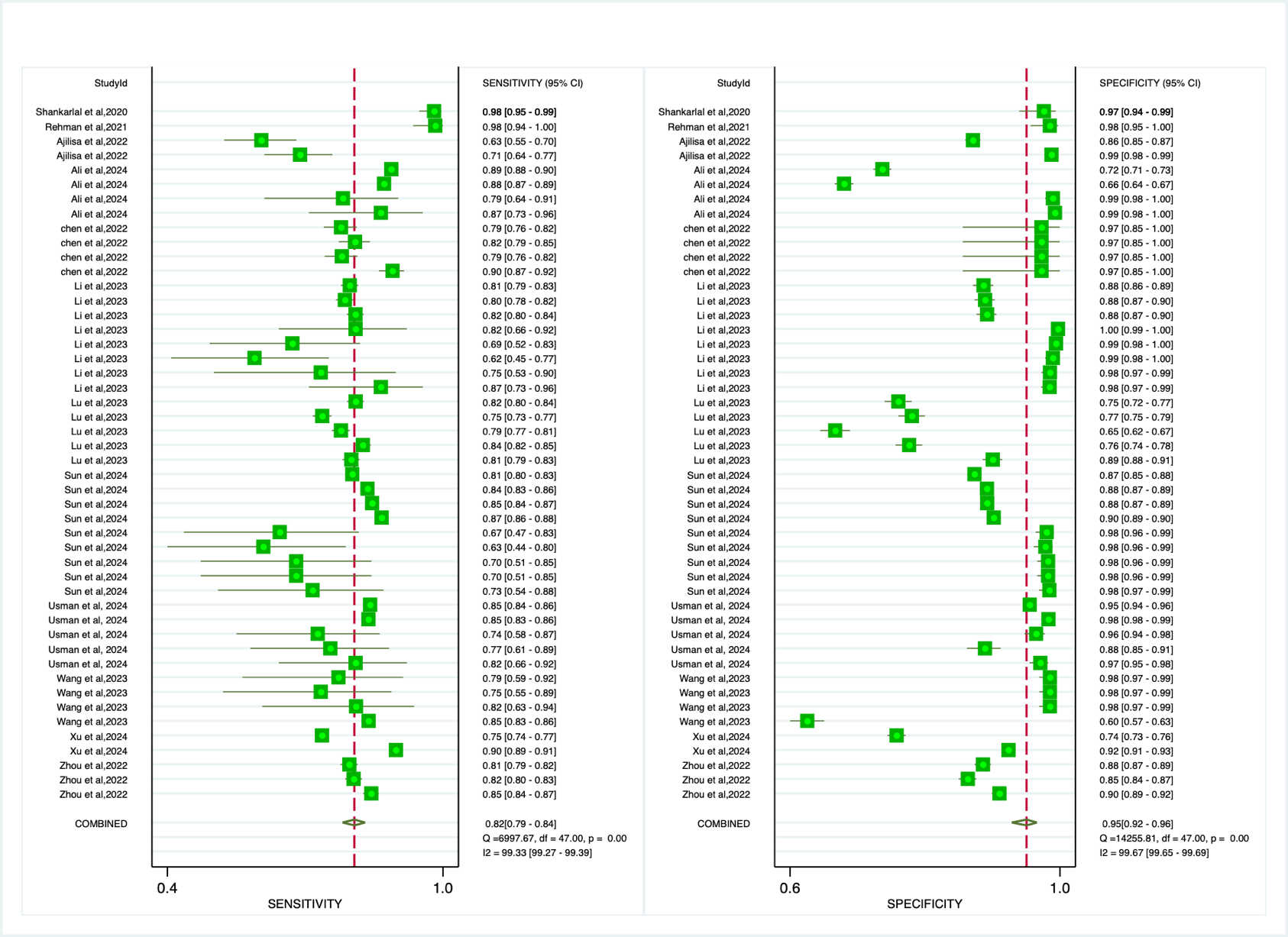


B.


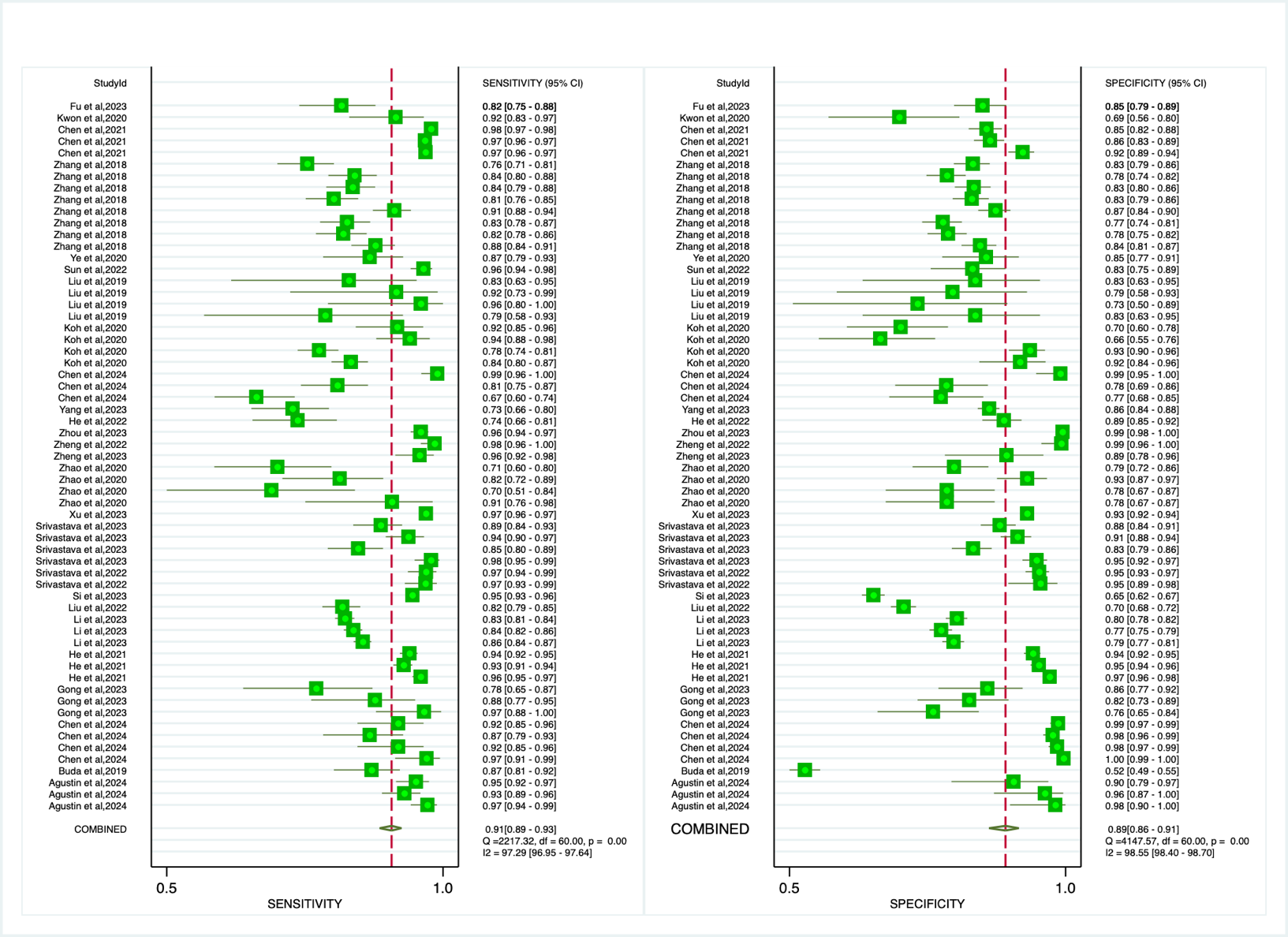

Supplement: Multimedia Appendix 6 [file jmir-v27-e73516-s006.docx]
